# Physical Activity Adherence Rates in Older Kidney Transplant Recipients: A Pilot Randomized Controlled Trial

**DOI:** 10.1093/geroni/igaa057.3263

**Published:** 2020-12-16

**Authors:** Tara O’Brien, Karen Rose, Alai Tan

**Affiliations:** The Ohio State University, Columbus, Ohio, United States

## Abstract

Daily walking activities are associated with improving cardiovascular and well-being in older kidney transplant recipients. Multicomponent interventions using technology and goal setting holds promise for sustaining daily walking activity among this population. The purpose of this randomized controlled trial pilot study was to evaluate the feasibility of a multicomponent intervention called SystemCHANGE™ + activity tracker for daily walking activity in older (age 60 and over) kidney recipients from baseline to 12 months. The intervention group implement a personal-system solution and wore a mobile activity tracker daily for 12 months. The attention-control group received educational information on healthy living as a transplant recipient and was asked to wear a mobile activity tracker daily for 12 months. Participants were randomized 1:1 to the intervention or control group. The sample consisted of 53 participants (n = 27 intervention, and n = 26 control). At the 12-month follow-up visit, the total study attrition rate was 23%. The adherence rates at 12 months were 96.5% in the intervention group and 80.8% in the attention- control group. The intervention group increased their steps from baseline to 12 months by 334 steps per day. The attention-control group demonstrated a decrease in steps by 563 steps per day. We found a mean difference of 1041± 2440 (Cohen’s d = 0.43) in daily steps between the groups from baseline to 12 months. The data suggests SystemCHANGE™ in combination with activity trackers may be feasible for older kidney transplant recipients to enhance and sustain physical activity with daily walking.

